# The Involvement of the Cryptococcal Yeast Kexin Protease in the Proteolytic Activation of the Uncleaved Influenza Virus Hemagglutinin Glycoprotein

**DOI:** 10.1002/mbo3.70384

**Published:** 2026-08-24

**Authors:** Nolwazi F. Ntshangase, Khwezi Mdana, Nozethu Mjokane, Winschau F. van Zyl, Jacobus Albertyn, Olihile M. Sebolai

**Affiliations:** ^1^ University of the Free State Bloemfontein South Africa; ^2^ Sol Plaatje University Kimberley South Africa

**Keywords:** *Cryptococcus neoformans*, furin protease, hemagglutinin (HA), influenza virus, mimetic peptide, protein–protein DOCKing (HADDOCK), proteolytic cleavage, yeast kexin protease (Kex2p)

## Abstract

Infectious diseases typically involve a single pathogen harming its host. However, in clinical settings, co‐infections also occur. This study investigated the potential interaction between *Cryptococcus* (*C*.) *neoformans* and influenza virus by assessing whether the cryptococcal yeast kexin protease (Kex2p) could activate the uncleaved hemagglutinin glycoprotein. Protein‐protein docking was performed using HADDOCK and independently validated using AlphaFold3. 6x His‐tagged truncated *KEX2* gene was expressed in *E. coli* BL 21 (DE3), and the recombinant protein was purified using nickel ion affinity chromatography. Successful protein production was confirmed by sodium dodecyl sulfate (SDS)‐polyacrylamide gel electrophoresis, anti‐His immunoblotting, and mass spectrometry. Recombinant Kex2p was evaluated in a biochemical peptide cleavage assay using a fluorogenic 12‐mer peptide mimicking the hemagglutinin HA1/HA2 site as substrate, with furin serving as the reference protease. HADDOCK predicted favorable binding of Kex2p to hemagglutinin (HADDOCK score = −119.2 ± 8.7; RMSD = 1.1 ± 0.2 Å), comparable to the furin‐hemagglutinin complex (HADDOCK score = −115.9 ± 5.7; RMSD = 2.1 ± 1.3 Å). AlphaFold 3 independently reproduced the HADDOCK‐predicted binding orientations, yielding moderate‐confidence complexes for hemagglutinin‐furin (ipTM = 0.50, pTM = 0.56; RMSD = 3.634 Å) and hemagglutinin‐Kex2p (ipTM = 0.41, pTM = 0.58; RMSD = 3.243 Å). In enzymatic assays, recombinant Kex2p exhibited a significantly higher Vmax than furin (*p* = 0.0411), indicating greater catalytic activity toward the hemagglutinin cleavage peptide. These findings provide the first biochemical and complementary computational evidence that *C. neoformans* Kex2p may recognize influenza hemagglutinin, supporting its potential role in hemagglutinin activation during cryptococcal‐influenza co‐infection.

## Graphical abstract

A haemagglutinin mimetic fluorogenic peptide assay revealed that recombinant Cryptococcus neoformans Kex2p exhibits strong haemagglutinin‐cleaving activity, suggesting a possible role for cryptococcal proteases in influenza virus activation.

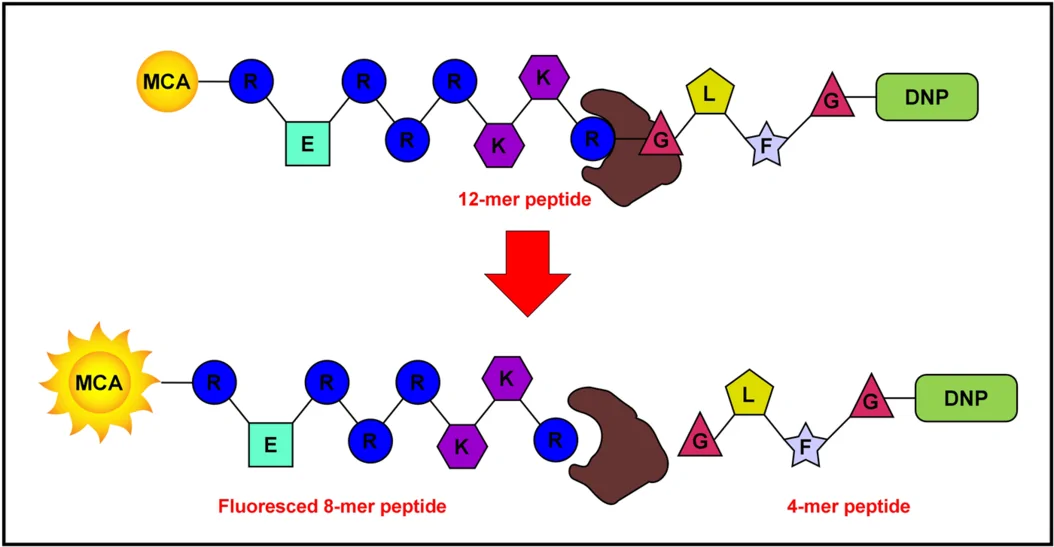

## 1 Introduction

Diverse communities of infectious agents can co‐exist in the lungs, resulting in different interactions (Gavillet et al. 2024). For example, the interacting pathogens and their microbial factors may work together in synergy to overburden a host (Salazar et al. 2022). Unfortunately, this could cause severe complications in patients, in turn worsening their outcomes. Of interest is the potential interaction that could occur between a respiratory virus like the influenza virus and *Cryptococcus neoformans*, a pneumonia‐causing pathogen.

*C. neoformans* is an important fungal pathogen that was recently included in the Critical Group of the World Health Organization's Fungal Priority Pathogens List (World Health Organization 2022). This organism is well endowed with hydrolytic enzymes, which are reported to be essential for fungal cellular growth and development, including virulence (Gutierrez‐Gongora and Geddes‐McAlister 2022). According to the MEROPS classification system, yeast kexin proteases are subtilisin‐like endopeptidases (Rawlings et al. 2018), similar to furin, their mammalian homolog, form part of the family S8 (Nakayama 1997). The role of these proteases in fungi was established after the characterization of deletion mutants, which were noted to lack the protease processing activity, among others. For example, in *Aspergillus niger*, the disruption of the kexin protease gene results in defects in the cell wall (van Leeuwe et al. 2020), increased sensitivity towards antifungal drugs that target the cell wall/cell membrane of *Candida glabrata* (Bader et al. 2008). *C. neoformans* has been shown to harbor a single gene coding for the Golgi‐associated Kex2p, which was reported on by Clarke et al. (2016) in their genome search. Currently, there is no direct evidence that this enzyme is extracellularly secreted.

While this pathogen can outright cause infections on its own (Bicanic and Harrison 2004), it has also been associated with viral co‐infections, as evidenced by case report studies involving the influenza virus (Hosseinnezhad and Rapose 2012; Gupta et al. 2015). For this virus to enter a host cell, its hemagglutinin (HA) glycoprotein, which appears as a spike on the virus surface, must become activated. Thus, to become fusion‐competent, HA must be cleaved by a host protease into two subunits, HA1 and HA2. This proteolytic cleavage exposes the otherwise hidden fusion peptide within HA2. Once cleaved, the HA1 binds to sialic acid‐containing receptors on the host cell surface, while the HA2 mediates virion‐cell fusion (To et al. 2013). The subsequent merging of the viral envelope with the endosomal membrane permits the release of the viral genome into the host cell's cytoplasm, thereby initiating the infection process (Tosheva et al. 2023). A critical aspect of HA activation lies in the precise nature of the cleavage site. This site is located at the interface between the HA1 and HA2 subunits within the HA0 precursor (To et al. 2013). In many influenza strains, the cleavage site is characterized by a monobasic motif, a single basic amino acid such as arginine, which is specifically recognized by endopeptidases secreted by host cells. However, in highly pathogenic strains, especially certain H5 and H7 subtypes, the cleavage site evolves to incorporate multiple basic amino acids, creating a polybasic cleavage site (To et al. 2013). This alteration allows a broader range of proteases, including ubiquitous enzymes like furin, to cleave HA, thereby expanding the range of tissues the virus can infect and increasing its pathogenicity.

We theorize that during a co‐infection, *C. neoformans* could contribute Kex2p, thus adding to a broad pool of furin‐like proteases that may be accessed and distorted by the influenza virus to activate its uncleaved fusogenic glycoprotein to initiate host entry. To this end, the current study sought to establish if cryptococcal Kex2p could cleave the influenza virus hemagglutinin glycoprotein through using the computational tool High Ambiguity Driven protein–protein DOCKing (HADDOCK). It is important to note that while this tool can be used to predict protein‐to‐protein complex structures based on biochemical and or biophysical information (Dominguez et al. 2003), it has some limitations, such as simplified representations of protein‐to‐protein interactions that often overlook dynamic processes that govern binding affinity and specificity (Warren et al. 2006). To address these limitations, it is essential to complement HADDOCK predictions with experimental validation. To this end, our in silico data was supported by a biochemical peptide cleavage assay.

## 2 Materials and Methods

### 2.1 Analyzing the Theoretical Activation of HA by the Cryptococcal Kex2p

The protocol was modified from a paper by Mjokane et al (Mjokane et al. 2024). In brief, molecular docking studies were conducted using X‐ray structures of Influenza virus hemagglutinin (PDB ID: 6PD5), human furin protease (PDB ID: 5JXH). These were obtained from the Research Collaboratory for Structural Bioinformatics (RCSB) and Protein Data Bank (PDB). The 3D structure of cryptococcal Kex2p, encoded by the gene CNAG_05446, was obtained as a predicted model from UniProt (accession number J9W1J0), as no experimentally determined structure is available. The structures were then optimized by removing water molecules and other chains that were not important (Aribisala et al. 2022). Molecular docking of hemagglutinin (target, modeled as a monomer) with furin (ligand) or cryptococcal Kex2p (ligand) was performed using the HADDOCK version 2.4 server with default parameters (Tosheva et al. 2023), analyzing protein–protein interactions through HADDOCK score, RMSD, van der Waals energy, electrostatic energy, desolvation energy, restraints violation energy, and buried surface area.

To complement the rigid‐body protein‐protein docking analyses performed using HADDOCK, AlphaFold 3 (AF3) was employed to predict the structures of the furin‐hemagglutinin and Kex2p‐hemagglutinin complexes independently. AF3 predicts protein complexes using an end‐to‐end deep learning framework that simultaneously models protein folding and intermolecular interactions (Abramson et al. 2024). The amino acid sequences corresponding to influenza virus hemagglutinin (PDB ID: 6PD5), furin protease (PDB ID: 5JXH), and *C. neoformans* Kex2p (UniProt accession: J9W1J0) were submitted to AlphaFold Server (https://alphafoldserver.com) as paired protein sequences, using a random seed of three while all other parameters were maintained at their default settings. The predicted complexes were visualized and structurally superimposed using UCSF Chimera version 1.19 (Pettersen et al. 2004).

### 2.2 Analyzing the Biochemical Activation of HA by the Cryptococcal Kex2p

#### 2.2.1 Preparation of the Start Material Required for the Biochemical Assay

##### 2.2.1.1 Isolation of the Cryptococcal Kex2p

To increase the sample amount of Kex2p to test in subsequent biochemical assays, it was decided to produce it using a scalable expression system such as *Escherichia coli*, as this enzyme is likely available at low endogenous levels. The *E. coli* expression approach is precedented in *Cryptococcus* literature (Ishibashi et al. 2012; Gressler et al. 2021), and importantly, *E. coli* is not known to encode for Kex2p. On the other hand, a yeast‐based expression system is not desired as yeasts such as *Saccharomyces cerevisiae* are known to also produce Kex2p (Redding et al. 1991). Our approach involved preparing a truncated version of the cryptococcal Kex2p to promote secretion into the extracellular environment. The truncation involved the removal of the signal peptide (1–20 amino acids) and amino acids at positions 661–900 on the N‐terminus, which are responsible for anchoring the protein in the membrane. These modifications allow for the protein to retain the functionality of the catalytic domain. In the end, the truncated protein had an expected molecular weight of 70 kDa, compared with 99 kDa for the full‐length protein. The DNA sequence was optimized for (2023). The sequence was subsequently cloned into the pET30a expression vector that contained a 6x His‐tag to facilitate protein isolation and purification. To facilitate isolation, a truncated Kex2p was expressed recombinantly in *E. coli* BL21 Star™ (DE3). This strain is widely used for recombinant protein production because of its rapid growth, ease of genetic manipulation, reduced protease activity and capacity for high‐level protein expression (Rosano et al. 2019). The vector was transformed into *E. coli* strain BL21 Star (DE3), and a single colony was selected for protein expression. The selected colony was then inoculated into an LB medium containing the kanamycin antibiotic and incubated at 37°C with shaking at 200 rpm. Protein expression was induced with isopropyl β‐D‐1‐thiogalactopyranoside (IPTG), while for large‐scale protein expression, recombinant BL21 Star (DE3) cells were inoculated into Terrific Broth (TB) medium containing kanamycin antibiotic and incubated at 37°C until the optical density (OD_600nm_) reached approximately 1.2 and protein expression was induced with IPTG at 15°C for 16 h. After incubation, cells were harvested (7025 *g*, 20 min at 4°C), then resuspended in a start buffer (SB, 50 mM Tris, 500 mM NaCl, 10% glycerol, pH 8.0) and sonicated using a one‐shot cell disruptor French‐Press. The supernatant was collected after centrifugation for 60 min at 15,870 *g* at 4°C for subsequent purification using nickel ion affinity chromatography. In brief, The His‐tagged Kex2p was loaded onto a 1.0 mL HisTrap HP column (GE Healthcare, PA, USA) and purified using nickel‐affinity chromatography with Ni^2+^‐NTA superflow resin (Thermo Fisher Scientific, IL, United States), following the manufacturer's instructions. Both the cell lysate and the Ni^2+^‐NTA resin were prepared with 10 mM imidazole in start buffer (SB10) before loading onto the column for purification on the ÄKTA purifier system (Amersham Biosciences, UK). The loaded column was washed with SB10, and proteins were eluted with a buffer containing 250 mM imidazole (SB250). Finally, the eluted His‐tagged proteins were desalted for optimal hNisP cleavage through anion exchange chromatography (Van Zyl et al. 2023). The protein concentration was determined by Bradford protein assay with bovine serum albumin (BSA) as the standard.

The protein separation pattern was assessed using the sodium dodecyl sulfate‐polyacrylamide gel electrophoresis (SDS‐PAGE). The SDS‐PAGE was prepared using the TGX FastCast Acrylamide Kit, 12% (Bio‐Rad, United States), according to the manufacturer's instructions. Protein samples were prepared by mixing with 1X Laemmli sample buffer (Bio‐Rad, United States) at a 1:1 ratio. Electrophoresis was then conducted, starting at 30 V to allow proteins to pass through the stacking gel, before increasing to 120 V once they entered the resolving gel. The run continued until the blue dye front reached the bottom of the glass plates, ensuring no sample loss due to migration off the gel.

The presence of the recombinant Kex2p was verified by Western blot for the crude sample. Here, following electrophoresis, the gel was transferred to a Polyvinylidene fluoride (PVDF) membrane. To facilitate efficient transfer, the membrane was initially soaked in methanol and then distilled water. The transfer apparatus was assembled with the membrane, fiber pads, and Whatman papers, all immersed in Transfer Buffer (3.03 g tris base, 14.4 g glycine, 200 mL methanol, 800 mL distilled water). Any air bubbles that formed were gently removed by rolling a glass tube over the membrane. Transfer proceeded at 100 V for 1–2 h at 4°C. Subsequent to transfer, the membrane underwent a series of optimization steps. First, it was stained with Ponceau S (Sigma‐Aldrich, United States) to verify protein transfer, then destained in water and rinsed with phosphate‐buffered saline (PBS) buffer (8.5 g NaCl, 1.4 g Na_2_HPO_4_, 0.2 g NaH_2_PO_4_, 1000 mL distilled water). Next, non‐specific binding sites were blocked with Blocking Buffer (100 mL PBS buffer, 5 g skim milk) for 1 h at 37°C. The membrane was then incubated overnight at 4°C with an anti‐His‐tag primary antibody (GenScript, The Netherlands), diluted in a Blocking Buffer. After rigorous washing, the membrane was treated with diluted HRP‐conjugated anti‐mouse secondary antibody for 1 h at 37°C. Finally, the ECL kit (Thermo Scientific, South Africa) was used according to the manufacturer's instructions, and the gel was visualized using ChemiDoc (Bio‐Rad, USA).

To complement the above, the crude sample was submitted to DIPLOMICS, Cape Town, South Africa, for mass spectrometry analysis. The submitted samples were analyzed using an internal protocol developed at DIPLOMICS. In preparation for protein concentration, Nanosep 3 K OMEGA filters (Merck, South Africa) were pacified in a 5% TWEEN20 (Merck, South Africa) solution overnight. The next morning, a total of three washes with deionised water were carried out to remove excess tween, followed by centrifuging 500 μL of deionised water at 10°C and 12,000 *g* for 20 min, and then repeating with 500 μL of 50 mM triethylammonium bicarbonate (TEAB; Merck, South Africa) to condition the filters. Samples were added to their respective Nanosep 3 K OMEGA filter tube and then centrifuged at 12,000 *g* until approx. A 50 μL of the sample was left above the filter. Samples were then made up to 60 μL using 2% Sodium dodecyl sulfate (SDS; Merck, South Africa) 50 mM TEAB. Quantification was conducted using the bicinchoninic acid (BCA) assay (Merck, South Africa) according to the manufacturer's instructions.

In preparation for the HILIC magnetic bead workflow, the HILIC beads (ReSyn Biosciences, South Africa) were aliquoted into a new tube, and the shipping solution was removed. Beads were then washed with 250 μL wash buffer (15% acetonitrile, 100 mM ammonium acetate (Merck, South Africa); pH 4.5) for one min. This was repeated once for a total of two washes. The beads were then resuspended in loading buffer (30% Acetonitrile, 200 mM ammonium acetate; pH 4.5) to a concentration of 2.5 mg/mL. A total of 20 μg from each sample was transferred to a protein LoBind plate (Merck, South Africa) and diluted with 50 mM TEAB to a final volume of 50 μL. HILIC magnetic beads were added at an equal volume to that of the sample, and a ratio of 5:1 total protein. The plate was then incubated at room temperature on the shaker at 900 rpm for 30 min for the binding of protein to beads. After binding, the beads were washed four times with 500 μL of 95% acetonitrile for 1 min. For digestion, Trypsin (Promega, South Africa), made up in 50 mM TEAB, was added at a ratio of 1:20 total protein, and LysC (Thermo Scientific, South Africa) was added at a ratio of 1:250 total protein. The plate was incubated at 45°C on the shaker for 2 h. After digestion, the supernatant containing peptides was then acidified by the addition of 5% FA to a final concentration of 0.5% FA. Approximately 150 ng of each sample was loaded onto an Evotip (Evosep Biosystems, Denmark). The loading process was carried out according to the manufacturer's instructions.

LCMS analysis was conducted with a timsTOF Pro 2 mass spectrometer (Bruker, United States) integrating trapped ion mobility (IM) separations coupled online via a Captivespray electrospray source (Bruker) to an Evosep One LC system (Evosep Biosystems, Denmark). Software for both systems was timsControl Setup Version: 5.0.9e3816ef3 and Bruker Compass HyStar 6.2 V6.2.1.13. Peptides were separated on a PepSep C18 column (150 μm × 8 cm, 1.5μm, Bruker, 1893470) maintained at 40°C. Separation was achieved using a pre‐formed linear gradient of solvents A and B (A: 0.1% FA; B: 100% acetonitrile/0.1% FA) over an 11‐min gradient (the standard 100SPD Evosep One method). The mass spectrometer was operated in positive ion mode. MS data was acquired using the standard “DDA PASEF‐ short gradient” DDA acquisition method (four PASEF ramps per cycle, 0.85–1.30 V‐s/cm2 CCS window, 100 ms TIMS window), precursor scans ranged from m/z 100 to 1700. The method duty cycle is estimated to be 0.53 s.

Data analysis was performed with MaxQuant (version 1.6.14.0) software using a *Cryptococcus neoformans* proteome (Cryptococcus_neoformans_6743prot_UP000002149) downloaded from UniprotKB on 05/04/2024. All processing parameters were kept as standard unless otherwise stated. The enzyme for digestion was set as trypsin. Deamidation of glutamine and asparagine, as well as oxidation of methionine, were specified as variable modifications with a maximum of five modifications per peptide. A fixed modification was added for the alkylation of cysteine by iodoacetamide (composition C(2)H(3)NO with a mass of 57.021464 Da).

##### 2.2.1.2 Preparation of the HA Mimetic Peptide

The amino acid sequence of the H5N1 influenza virus hemagglutinin glycoprotein was first obtained from the National Center for Biotechnology Information (NCBI) and analyzed using the prediction algorithm, namely the ProPeptide Cleavage Site Prediction (ProP). This is a bioinformatics resource that predicts furin proteolytic cleavage sites based on established consensus motifs (Stout et al. 2021). In brief, the tool assigns a score of between 0 and 1. A site with a score above 0.5 is predicted to be a furin cleavage site (Stout et al. 2021). It was established that the H5N1 influenza virus hemagglutinin protein gave a ProP score of 0.8, indicating a high likelihood of furin cleaving hemagglutinin (Table 1).

**Table 1:** The obtained ProP score used to predict the presence and location of furin cleavage sites in the H5N1 influenza virus HA protein.

Sequence	Position	ProP score
RERRRK**KR** ↓ GLFG	344	0.829

The amino acid sequence, that is, RERRRK**KR** ↓ GLFG, obtained after using the ProP prediction algorithm, containing the furin‐recognizable cleavage site, was submitted to Biomatik (Ontario, Canada) to prepare a synthetic 12‐mer hemagglutinin peptide that was modified to incorporate intramolecular quenching. The principle is that upon cleavage by commercial recombinant furin or cryptococcal recombinant Kex2p, the fluorescence of the fluorophore, namely, 7‐methoxycoumarin‐4‐yl acetyl, cannot be absorbed by a quencher, namely, N‐2,4‐dinitrophenyl, thus this fluorescence can be measured.

#### 2.2.2 Biochemical Peptide Cleavage Assay

A biochemical peptide cleavage assay based on the protocol of Warren et al. (2006) was modified and used to examine whether a commercial recombinant furin and cryptococcal recombinant Kex2p could cleave the prepared mimetic 12‐mer hemagglutinin peptide. The assay reaction mixture consisted of a 100 μl buffer solution (pH 7.5) comprising 100 mM HEPES, 0.5% Triton X‐100, 1 mM CaCl_2_, and 1 mM 2‐mercaptoethanol. These reaction conditions were reported to be optimal for the hydrolysis of a substrate by furin (Izidoro et al. 2009). To initiate catalysis, 0.5 μL of recombinant furin (10 U/mL; New England Biolabs, USA) was added to the reaction mixture. In a separate experiment, 0.5 μL of a crude Kex2p sample was used.

Reactions were performed at 30°C, and fluorescence emission was measured every minute for 45 min using a fluorometer with excitation (λ355 nm) and emission (λ405 nm) wavelength settings. Six independent experiments were conducted, and the mean Vmax was calculated. A negative control consisting of buffer only and a peptide control consisting of buffer with peptide was included to determine the background signal for normalization. The Michaelis–Menten plot and the Lineweaver–Burk plot were plotted to assess the relationship between a specific protease sample, that is, commercial recombinant furin or cryptococcal recombinant Kex2p (crude sample) and the mimetic hemagglutinin peptide as the substrate.

### 2.3 Statistical Analyses

For each study, unless otherwise stated, three individual independent experiments were performed. For each of the individual experiments, no technical repeats were done. GraphPad Prism was used to calculate the statistical significance of the experimental data using an unpaired *t*‐test. Mean values and standard error of the means (SEM) were calculated, and the statistical significance of the data was considered at *p* < 0.05.

## 3 Results

### 3.1 Cryptococcal Kex2p Can Theoretically Interact With Hemagglutinin

**Figure 1:**
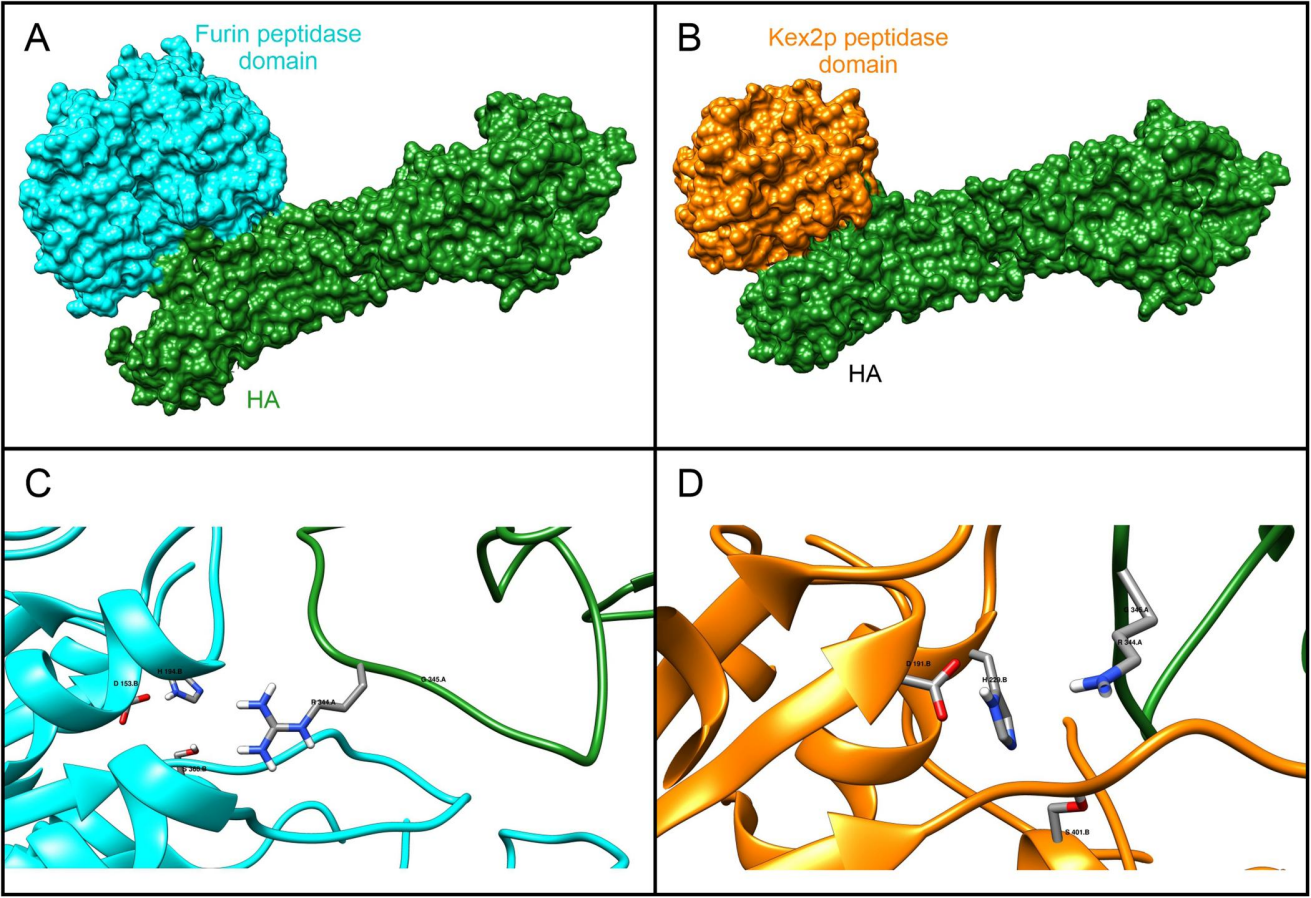
The surface representation of the docked (A) human furin peptidase domain‐HA complex and (B) cryptococcal Kex2p peptidase domain‐HA complex. The cleavage site, although not fully exposed, is at the interface between the enzymes (furin and Kex2p) and the substrate (HA monomer). The intermolecular interactions of the DOCKed human furin peptidase domain–HA complex (C) and cryptococcal Kex2p peptidase domain–HA complex (D), showing the three‐dimensional spatial arrangement of serine, histidine and aspartate in the active site region in relation to the scissile peptide bond (R344) on the HA peptide. In (A), blue represents the furin peptidase domain, and green depicts the docked HA monomer, while in (B), orange is the Kex2p peptidase and green is the docked HA monomer. In (C), blue represents the furin and green depicts the HA, while in (D), orange is the Ke2p and green is the HA. HA, hemagglutinin; Kex2p, kexin protease.

In this study, HADDOCK was used for protein–protein docking due to its strong performance in predicting interactions. Table 2 shows the obtained results after DOCKing furin and hemagglutinin, as well as *C. neoformans* Kex2p and hemagglutinin (H5N1). A HADDOCK score of −115.9 ± 5.7 was recorded when furin‐hemagglutinin was DOCKed. In contrast, the DOCKing of Kex2p‐hemagglutinin gave a score of −119.2 ± 8.7, which was comparable (*p* > 0.05) to that of furin‐hemagglutinin. The average RMSD value and Z‐score of furin in complex with hemagglutinin were 2.1 ± 1.3 Å and 2.0, and that of Kex2p were 1.1 ± 0.2 Å and −1.6. In docking, the degree of fitness of an entity at the binding domain of a protein is measured. Here, a high negative score is suggestive of better orientation and high affinity (Aribisala et al. 2022). The negative DOCKing scores of Kex2p and furin are indicative of their better fitness and orientation at the binding pocket of hemagglutinin. Furthermore, the RMSD measures the degree of stability of a system, and an RMSD value of less than 3 Å is generally good and within an acceptable limit to assess the stability of a resulting protein‐ligand/protein complex (Aribisala et al. 2022; Ramírez and Caballero 2018; Ferreira et al. 2015). Therefore, both the obtained RMSD values in this study were acceptable, while the Kex2p value suggested a stronger affinity with hemagglutinin than furin. The Van der Waals energy and electrostatic energy that contribute to the binding strength for each complex were calculated. For the furin‐hemagglutinin complex, the Van der Waals and electrostatic energy values were −39.5 ± 2.9 kcal/mol and −485.1 ± 44.7 kcal/mol, respectively. Compared to these, the Kex2p‐hemagglutinin complex had scores of −50.5 ± 6.3 kcal/mol (Van der Waals energy) and −365.6 ± 20.9 kcal/mol (electrostatic energy). The buried surface area, that is, the amount of protein surface not in contact with water upon complexation, data was calculated and analyzed. Here, it was noted that Kex2p had a score of 2056.1 ± 96.9 Å^2^ while that of the furin (1812.6 ± 53. 1 Å^2^). The furin's desolvation energy (15.5 ± 3.2 kcal/mol) and restraint violation energy (51.4 ± 17.2 kcal/mol) were observed to be far less than the buried surface area score (1812.6 ± 53. 1 Å^2^). Similar readings were obtained for the Kex2p. This protease had a score desolvation energy of −4.3 ± 3.4 kcal/mol and restraint violation energy of 3.5 ± 3.5 kcal/mol. These values were, likewise, far less than the buried surface area score (2056.1 ± 96.9 Å^2^). In Figure 1A,B, information related to the surface representation of the docked complexes is provided, that is, furin‐hemagglutinin and Kex2p‐hemagglutinin, at their respective optimal orientation. Figure 1C,D further summarize the characteristic amino acid residues for each protease that were poised to interact with the hemagglutinin cleavage site (↓ R344) in the docked complex structure. Importantly, the coordinated amino acids of the catalytic triad for each ligand were seen, that is, for furin (D153, H194, and S 368) and for Kex2p (D191, H229, and S401).

**Table 2:** The intermolecular binding energies of the DOCKed human furin peptidase domain–HA complex and cryptococcal Kex2p peptidase domain–HA complex.

Protein pair	RMSD (Å)	HADDOCK score	Vander Waals energy (kcal/mol)	Electrostatic energy (kcal/mol)	Desolvation energy (kcal/mol)	Restraints violation energy (kcal/mol)	Buried surface area (Å^2^)	Z‐ score
Furin‐HA	2.1 ± 1.3	−115.9 ± 5.7	−39.5 ± 2.9	−485.1 ± 44.7	15.5 ± 3.2	51.4 ± 17.2	1812.6 ± 53.1	2.0
Kex2p‐HA	1.1 ± 0.2	−119.2 ± 8.7	−50.5 ± 6.3	−365.6 ± 20.9	−4.3 ± 3.4	3.5 ± 3.5	2056.4 ± 96.9	−1.6

Abbreviations: HA, hemagglutinin; HADDOCK, protein–protein DOCKing; Kex2p, kexin protease; RMSD, root mean square deviation.

**Figure 2:**
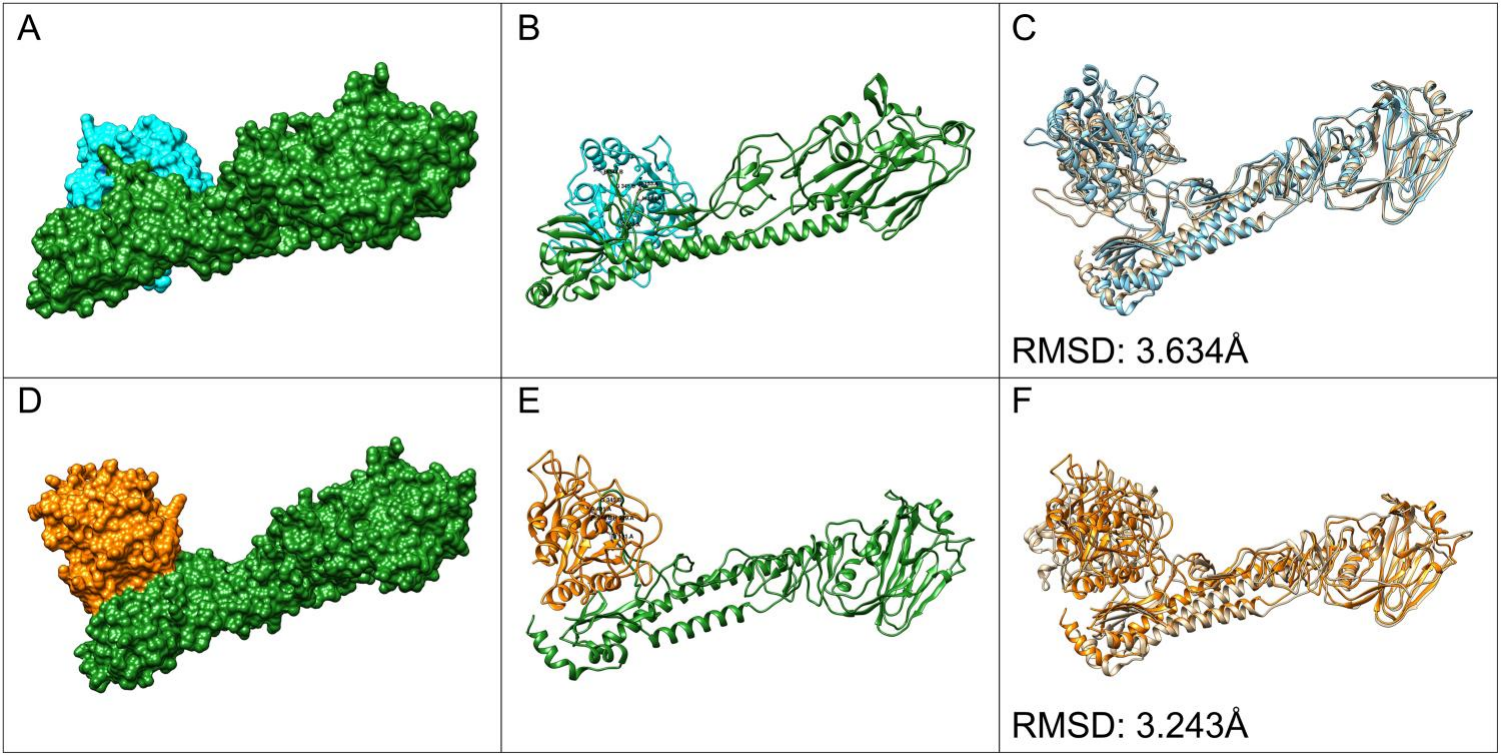
Molecular docking models showing the predicted interactions between the HA, furin and Kex2p using AlphaFold3: (A) HA–furin surface representation, (B) Intermolecular interaction of HA and furin highlighting the catalytic triad residues (C) the superimposed HADDOCK HA–furin complex with the AF3 HA–furin complex. And (D) HA–Kex2p surface representation, (E) Intermolecular interaction of HA and Kex2p highlighting the catalytic triad residues, (F) the superimposed HADDOCK HA‐Kex2p complex with the AF3 HA–Kex2p complex. In A and B, the blue represents the furin peptidase domain, and green depicts the docked HA monomer. In C, brown is the HADDOCK HA–furin complex, and blue is the AF3 HA–furin complex. In D and E, the orange represents the Kex2p peptidase domain, and green depicts the docked HA monomer. In F, brown is the HADDOCK HA–Kex2p complex, and orange is the AF3 HA–Kex2p complex., while in B, orange is the Kex2p peptidase and green is the docked HA monomer. In C, blue represents the furin and green depicts the HA, while in D, orange is the Ke2p and green is the HA. HA, hemagglutinin; HADDOCK, protein–protein DOCKing; Kex2p, kexin protease.

The protein‐protein interactions predicted by HADDOCK were independently evaluated using AF3 (Table 3 and Figure 2). The AF3‐predicted complexes exhibited binding orientations comparable to those generated by HADDOCK, with both furin and Kex2p occupying the same region of the hemagglutinin surface as observed in the HADDOCK models. Furthermore, several of the interacting residues identified in the HADDOCK complexes were conserved in the AF3‐predicted complexes, indicating similar interaction interfaces between the two computational approaches. Together, these observations support the structural plausibility of the predicted hemagglutinin‐protease (furin or Kex2p) interactions.

**Table 3:** AlphaFold3 confidence matrices and structural comparison with HADDOCK‐predicted complexes.

Protein complex	ipTM	pTM	RMSD (Å)
Furin–hemagglutinin	0.50	0.56	3.634
Kex2p–hemagglutinin	0.41	0.58	3.243

Abbreviations: HADDOCK, protein–protein DOCKing; ipTM, interface predicted Template Modelling; Kex2p, kexin protease; pTM, predicted Template Modelling; RMSD, root mean square deviation.

Structural superimposition of the HADDOCK and AF3 complexes yielded RMSD values of 3.634 Å for the hemagglutinin‐furin complex and 3.243 Å for the hemagglutinin–Kex2p complex, indicating moderate structural agreement between the independently generated models. The AF3 prediction for the hemagglutinin–furin complex produced an interface predicted Template Modelling (ipTM) score of 0.50 and a predicted Template Modelling (pTM) score of 0.56, whereas the hemagglutinin–Kex2p complex yielded an ipTM score of 0.41 and pTM score of 0.58. These confidence metrics suggest moderate confidence in the predicted interfaces, while supporting the overall structural arrangement of both complexes. Collectively, the agreement between HADDOCK and AF3 provides independent computational support for the proposed interaction between hemagglutinin and the two proteases.

### 3.2 Cryptococcal Kex2p Mediates the Proteolytic Cleavage of Hemagglutinin

**Figure 3:**
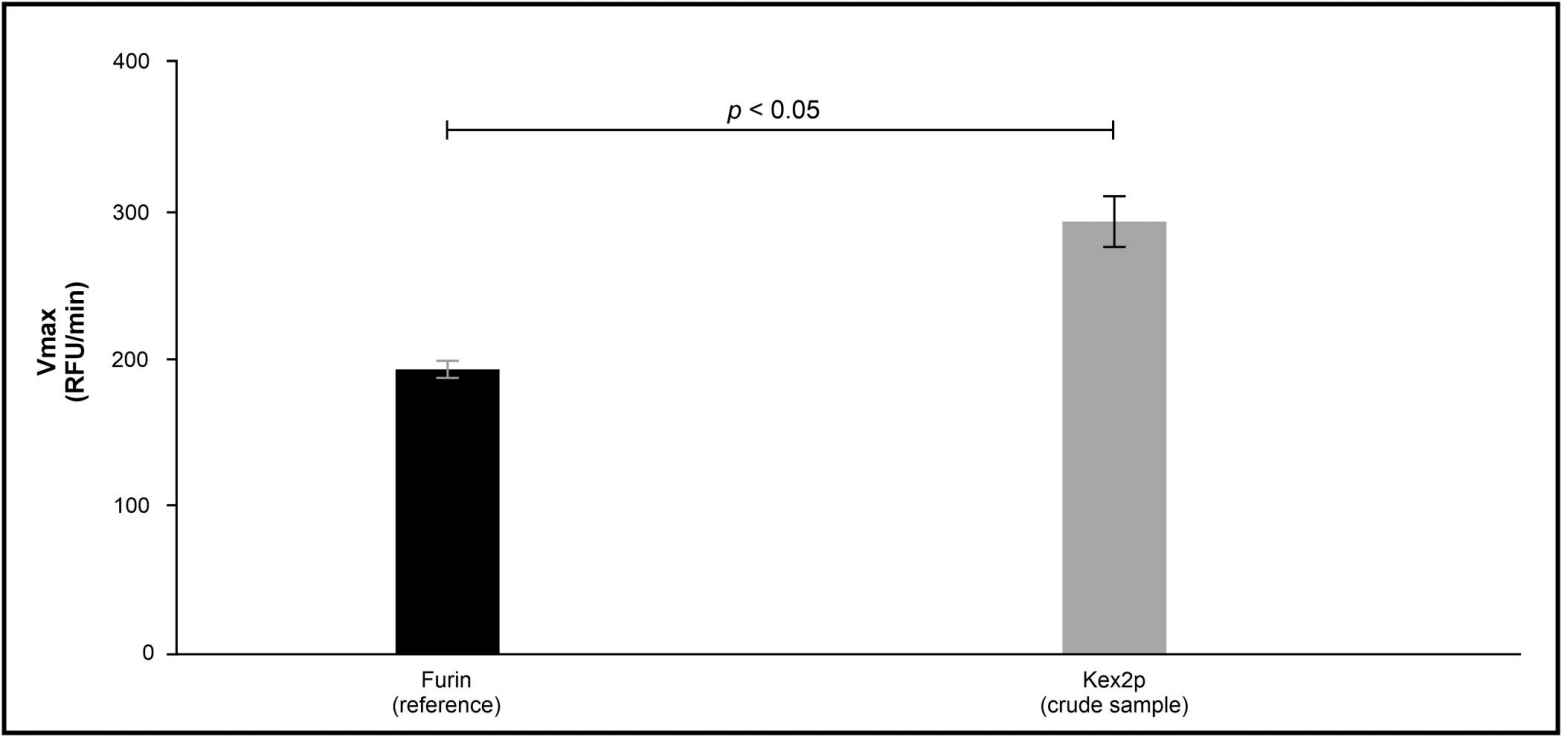
The blotted membrane that was probed with an anti‐His‐tag primary antibody to immunodetect the presence of Kex2p in the crude sample. M = Marker; Crude Kex2p = crude sample containing the cryptococcal recombinant Kex2p. The red block highlights the position of the band of interest, that is, Kex2p, on the gel with a size of approximately 70 kDa. Kex2p, kexin protease.

It was first sought to confirm if Kex2p was successfully isolated. The image showing the blotted membrane is presented in Figure 3. A distinct immunodetected band of approximately 70 kDa is observed on a lane that was loaded with the crude sample. This band corresponded to the expected molecular weight of the truncated Kex2p. The detection of this band in the crude sample confirmed the successful heterologous expression of Kex2p in the *E. coli* BL21 (DE3) cells. To confirm if indeed this sample contained Kex2p, mass spectrometry analysis was performed. The mass spectrometry data showing the peptide information associated with cryptococcal Kex2p is summarized in Table 4. The mass spectrum for each of the identified peptides with a B‐ and Y‐ion series, showing where cleavage occurred along the peptide backbone, is depicted in Supporting Information: Figures S1 and S2. The masses of the B‐ and Y‐ions were used to deduce the sequence of the amino acids within the peptide.

**Table 4:** Identification of peptides associated with cryptococcal Kex2p detected using LC‐MS.

Identified serine protease	Number of peptides associated with Kex2p	Characterisation of the identified peptides				
		Peptide lengths	Peptide range	Peptide sequence	Mass error	Mass (M + H)^+^
Cryptococcal Kex2p	2	49	179–227	R.GVTGEGVHVVIIDDGLDVESKDLKDNFFAEGSYDFNDHTALPIPR.L	1.2	4901.390
		43	462–504	K.LDAGLFVEAAEKWELVKPQTWYDSPAVYLPTTSPADATK.R	2.6	4308.175

Abbreviations: Kex2p, kexin protease; LC‐MS, liquid chromatography‐mass spectrometry.

**Figure 4:**
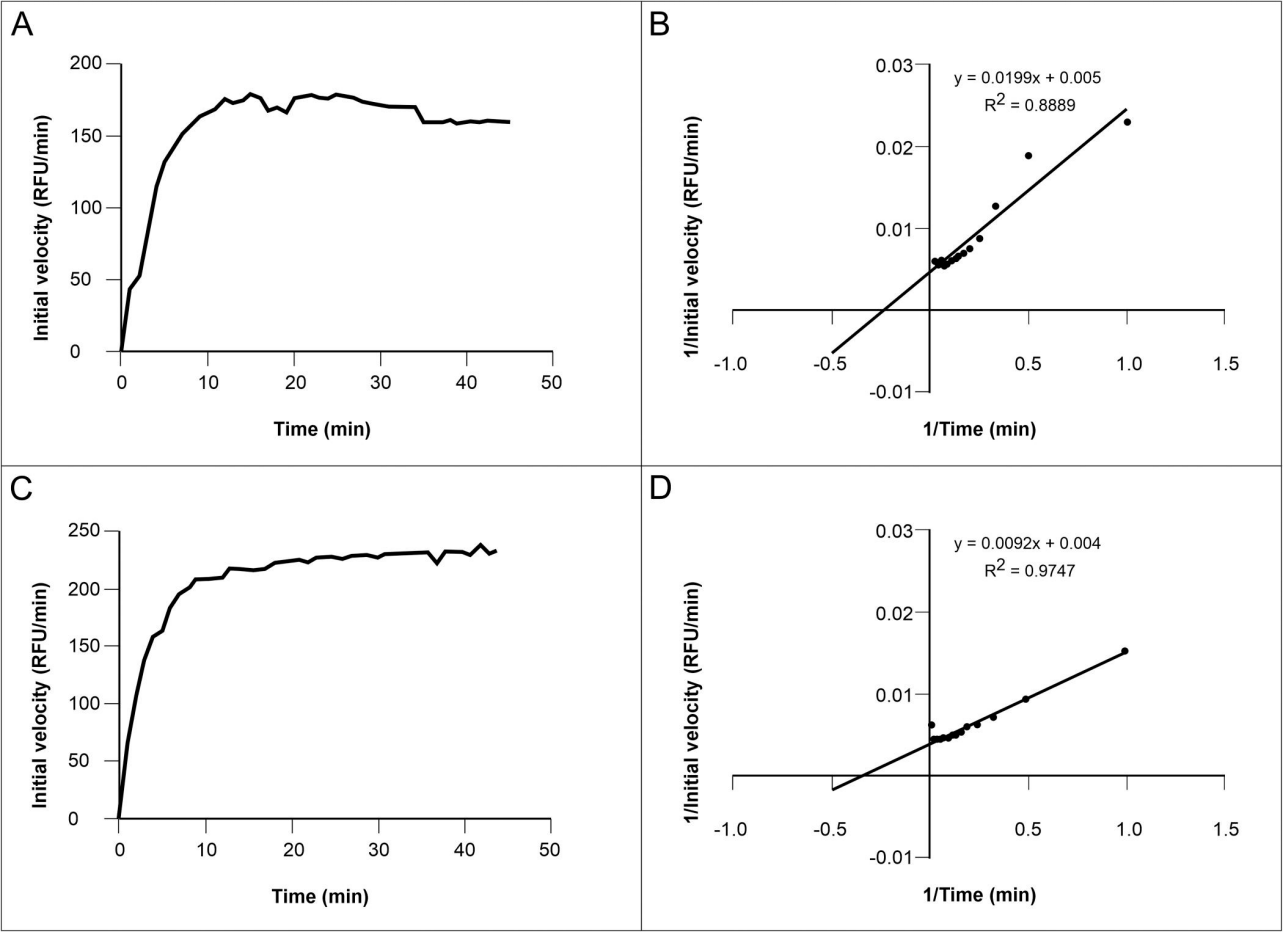
(A) The depiction of recombinant furin‐catalyzed reaction velocity of the substrate, i.e., hemagglutinin mimetic peptide over time (45 min) for one of the independent experiments. (B) The Lineweaver–Burk transformation graph of the Michaelis–Menten equation, plotting 1/velocity against 1/time (min) for one of the independent experiments. (C) The depiction of Kex2p‐catalyzed reaction velocity of the substrate, i.e., hemagglutinin mimetic peptide over time (45 min) for one of the independent experiments. (D) The Lineweaver–Burk transformation graph of the Michaelis–Menten equation, plotting 1/velocity against 1/time (min) for one of the independent experiments. These results provide insight into the cleavage efficiency of the hemagglutinin mimetic peptide during the recombinant furin‐catalyzed reaction and Kex2p‐catalyzed reaction. Kex2p, kexin protease.

The measure of the maximum rate at which the recombinant furin (positive control sample) and recombinant Kex2p (test sample) could catalyze the proteolysis reaction of the mimetic hemagglutinin under optimal conditions was recorded (Figure 4 and Table 5). As expected, the recombinant furin could cleave the mimetic peptide, and thus, its calculated Vmax was used as a reference reading. When considering the reading obtained for the recombinant Kex2p, it was noted that the calculated Vmax was significantly higher than that of the recombinant furin (*p* = 0.0411). The latter suggested that the recombinant Kex2p displayed proteolytic activity sufficient to cleave the mimetic peptide. However, a limitation of the data is the lack of a protease inhibitor, including other substrates without a multibasic motif.

**Table 5:** Calculated Vmax values for recombinant *Cryptococcus neoformans* Kex2p and furin during cleavage of an influenza hemagglutinin‐derived fluorogenic peptide substrate.

Protein complex	Calculated Vmax (RFU/min)					
	Experiment 1	Experiment 2	Experiment 3	Mean	SEM	*p*‐value
Furin–hemagglutinin	200	167	200	189	11	0.0411
Kex2p–hemagglutinin	250	250	333.33	277.67	27.67	

Abbreviations: Kex2p, kexin protease; SEM, standard error of mean.

## 4 Discussion

**Figure 5:**
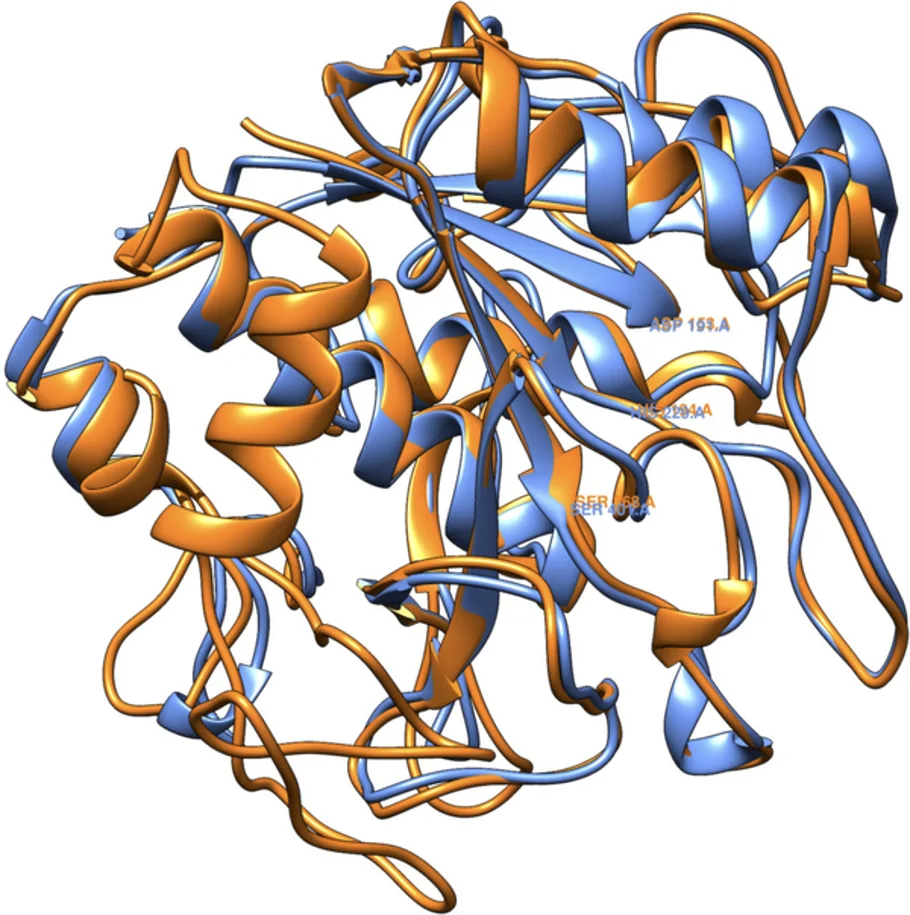
The superimposed peptidase domain structures of the human furin and *Cryptococcus neoformans* Kex2p proteases (RMSD = 0.999 Å), highlighting the conserved catalytic triad. The alignment demonstrates that all key catalytic residues, serine, histidine, and aspartate, occupy identical positions in both proteases. Moreover, the amino acid sequence similarity between the two enzymes was 25.52%, indicating structural and functional conservation between the two. RMSD, root mean square deviation.

While the manifestation of a viral infection can bring about an out‐of‐control inflammatory response that may create optimal conditions for a fungal infection to develop (Redding et al. 1991), it is unclear if a fungal infection could also precipitate a viral infection. To this end, the current paper attempted to elucidate whether a microbial factor, such as Kex2p, could play a role in activating the fusogenic glycoprotein of a virus, leading to its invasion. The interest in Kex2p was informed by the evolutionary relationship that exists between Kex2p and furin. Literature documents that these two proteases are proprotein convertases (Fuller et al. 1989), which share structural similarity with a percentage identity of 25.52%, which translates into functional homology and an RMSD of 0.999 Å was obtained (Figure 5).

Therefore, by implication, it is reasonable to foresee cryptococcal Kex2p, similar to it furin, processing substrates that bear the multibasic motifs of the preferred consensus sequence **R**‐**R** or **R**‐**K** ↓ . To illustrate this, Bresnahan and co‐workers showed that Kex2p could process a precursor of the mouse nerve growth factor into a competent protein that is documented to regulate sensory neuron growth and development (Bresnahan et al. 1990). While Foster and co‐workers reported that Kex2p could process the human protein C precursor into a mature protein C (Foster et al. 1991), which regulates coagulation and inflammation (Griffin et al. 2018).

In this study, the substrate of interest was hemagglutinin of the influenza virus, which requires an exogenous endopeptidase for its proteolytic activation. We presented in silico data, wherein HADDOCK and AF3 were used to investigate the interaction of hemagglutinin with furin and cryptococcal Kex2p. The HADDOCK program has successfully been used elsewhere to show the molecular mechanism of furin in activating viral substrates such as SARS‐CoV‐2 (Ho et al. 2023), while AF3 and AlphaFold‐Multimer frequently reproduce correct global protein complex conformations even when interface confidence is moderate (Abramson et al. 2024).

Although these approaches employ fundamentally different modelling strategies, they converged on comparable binding orientations, with both proteases occupying the same regions of the hemagglutinin molecule and sharing several conserved interacting residues. The agreement observed between HADDOCK and AF3 models strengthens the computational evidence that cryptococcal Kex2p may recognize influenza hemagglutinin in a manner analogous to human furin.

Nevertheless, these findings remain computational predictions and required experimental validation using a complementary biochemical approach to confirm the biological relevance of the proposed interactions. For this purpose, a mimetic peptide resembling the hemagglutinin glycoprotein at the HA1/HA2 site was used. Mimetic peptides are synthetically engineered peptides, consisting of 5–20 amino acids, designed to mimic the structural and functional properties of their native counterparts and could be used to study their interaction with specific receptors or molecules (Groß et al. 2016). It was possible, in the current study, to show that the cryptococcal Kex2p could indeed proteolytically cleave the mimetic hemagglutinin. This observation is, in part, in line with the data reported by Straus and Whittaker (2017), wherein they showed that various soluble proteases, including furin and trypsin, could cleave influenza viral hemagglutinin protein. One of the limitations of using mimetic peptides is that they have a short sequence of amino acids, which may not fully replicate the complexity and functionality of a full‐length virus protein.

While these findings suggest a possible interaction in the context of influenza virus co‐infection with cryptococcal infections, the in vivo relevance and physiological concentration of secreted Kex2p in the lung remain unknown. Therefore, the potential for viral activation in vivo requires further investigation. There is currently no direct evidence that Kex2p is secreted extracellularly; thus, its potential behavior is inferred from studies of its mammalian homolog, furin. Under hypoxic conditions, furin has been shown to translocate from the Golgi apparatus to the cell surface, where it mediates surface‐level activation of substrates (Arsenault et al. 2012). Such conditions are relevant in vivo, particularly in the context of a cryptococcal disease. To expand on this, following lung colonization, fungal cells become localized within immune cell‐rich alveolar environments and are subsequently contained in the granulomatous lesions. These structures impose host‐driven constraints that limit oxygen availability, thus exposing this organism to sustained or intermittent hypoxic (Telzrow et al. 2022). Consistent with this, studies in tuberculosis have established that granulomas are intrinsically hypoxic microenvironments that exert selective pressure on microbial physiology (Datta et al. 2016; Qualls and Murray 2016). By implication, such hypoxic conditions may similarly influence Kex2p localization and function during infection.

In conclusion, to our knowledge, this is the first empirical in vitro data showing the potential of cryptococcal Kex2p in activating the influenza hemagglutinin. It is now prudent to confirm the data using pseudovirions, as they can be engineered to have the full‐length peptide of the desired fusogenic glycoprotein. The clinical relevance of this data is that, in the context of the influenza virus co‐infecting a host with an underlying cryptococcal infection, this virus could, in addition to accessing furin, access the cryptococcal Kex2p and use it to achieve the activation of its fusogenic glycoprotein, leading to viral invasion and replication. Therefore, to control this unwanted proteolysis, it is crucial to clear underlying fungal infections. This may also be completed by using a Kex2p inhibitor to regulate the Kex2p‐uncleaved glycoprotein interaction, including the furin‐uncleaved glycoprotein interaction.

## Author Contributions

**Nolwazi F. Ntshangase:** methodology, investigation, formal analysis, writing – original draft. **Khwezi Mdana:** investigation, formal analysis, writing – review and editing. **Nozethu Mjokane:** methodology, writing – review and editing. **Winschau F. van Zyl:** methodology, resources, writing – review and editing. **Jacobus Albertyn:** supervision, writing – review and editing. **Olihile M. Sebolai:** conceptualization, funding acquisition, project administration, resources, supervision, writing – review and editing.

## Ethics Statement

The authors have nothing to report.

## Conflicts of Interest

The authors declare no conflicts of interest.

## Supporting information


**Figure S1:** The obtained MS/MS spectrum of the peptide R.GVTGEGVHVVIIDDGLDVESKDLKDNFFAEGSYDFNDHTALPIPR.L associated with cryptococcal Kex2p showing a unique fragmentation pattern. The B‐ions represented the fragments retaining the N‐terminus charge, while the Y‐ions carry the charge on the C‐terminal side. This peptide was determined to have a mass‐to‐charge ratio of 4901.

**Figure S2:** The obtained MS/MS spectrum of the peptide K.LDAGLFVEAAEKWELVKPQTWYDSPAVYLPTTSPADATK.R associated with cryptococcal Kex2p showing a unique fragmentation pattern. The B‐ions represented the fragments retaining the N‐terminus charge, while the Y‐ions carry the charge on the C‐terminal side. This peptide was determined to have a mass‐to‐charge ratio of 4308.

## Data Availability

Data sharing not applicable to this article as no datasets were generated or analyzed during the current study.
